# Integrating Food Safety, Nutrition Security, and Sustainability Indicators for Food System Monitoring in Low- and Middle-Income Countries: A Structured Methodological Review and Framework Development Study

**DOI:** 10.3390/foods15152658

**Published:** 2026-07-28

**Authors:** Lara Hanna-Wakim, Yonna Sacre, Maha Hoteit

**Affiliations:** 1 Department of Agricultural and Food Engineering, School of Engineering, Holy Spirit University of Kaslik (USEK), P.O. Box 446, Jounieh 1200, Lebanon; 2 Department of Nutrition and Food Sciences, Faculty of Arts and Sciences, Holy Spirit University of Kaslik (USEK), P.O. Box 446, Jounieh 1200, Lebanon; yonnasacre@usek.edu.lb; 3 PHENOL Research Program, Faculty of Public Health, Section 1, Lebanese University, Beirut P.O. Box 6573, Lebanon; m.hoteit@ul.edu.lb; 4 Department of Primary Care and Population Health, University of Nicosia Medical School, Nicosia P.O. Box 24005, Cyprus; 5 INSPECT-LB (Institut National de Santé Publique, d’Épidémiologie Clinique et de Toxicologie-Liban), Beirut 1103, Lebanon

**Keywords:** food safety, nutrition security, sustainable food systems, one health, food system monitoring, indicators, low- and middle-income countries (LMICs), food security, sustainability, food governance

## Abstract

Access to safe, nutritious, and sustainable food remains a major challenge in many Low- and Middle-Income Countries (LMICs), where food insecurity, malnutrition, and foodborne diseases frequently coexist. Despite growing recognition of the interconnections between food safety, nutrition security, and sustainability, monitoring systems often assess these dimensions separately, limiting the effectiveness of policy interventions. This study conducted a structured methodological review and framework development study of indicators used to monitor food safety, nutrition security, and sustainability within food systems, with particular emphasis on LMIC contexts. Peer-reviewed literature and internationally recognized monitoring frameworks, drawn from Scopus, Web of Science, PubMed, Google Scholar, and institutional repositories including FAO, WHO, World Bank, and GAIN, were systematically reviewed, yielding 75 sources encompassing 54 peer-reviewed articles and 21 institutional reports, from which 47 indicators were identified and assessed. Indicators were evaluated according to relevance, data availability, methodological robustness, and policy actionability. The review identified a core set of ten operationally relevant indicators, including the Global Diet Quality Score (GDQS), Minimum Dietary Diversity for Women (MDD-W), Food Insecurity Experience Scale (FIES), foodborne disease burden indicators, cost of a healthy diet, and post-harvest loss rates. Building on these findings, an integrated indicator selection framework is proposed to support context-specific monitoring and decision-making in LMICs. The results suggest the value of a One Health approach that links human, animal, and environmental health, and three One Health interface indicators, antimicrobial resistance prevalence in food chain isolates, zoonotic disease incidence attributable to food, and pesticide residue exceedance rate in fresh produce, are explicitly integrated into the proposed framework with LMIC-specific implementation pathways. A key limitation of this review is that it did not employ formal systematic methods and may not have exhaustively identified all relevant indicators across the full breadth of the published literature.

## 1. Introduction

Achieving universal access to safe, nutritious, and sustainable food remains one of the most pressing global challenges. Food security exists when all people, at all times, have physical and economic access to sufficient, safe, and nutritious food that meets their dietary needs and food preferences for an active and healthy life [1]. Despite considerable progress in agricultural productivity and food availability, food insecurity, malnutrition, and foodborne diseases continue to impose substantial health, social, and economic burdens worldwide [2,3,4]. Globally, an estimated 600 million people fall ill each year due to contaminated food, resulting in approximately 420,000 deaths and 33 million disability-adjusted life years (DALYs) lost annually [2,3].

The intersection between food safety and nutrition security is particularly important in Low- and Middle-Income Countries (LMICs), where unsafe food, inadequate diets, and environmental pressures frequently coexist. Foodborne illnesses can impair nutrient absorption, increase metabolic demands, and contribute to stunting, wasting, and micronutrient deficiencies, particularly among children under five years of age [5]. At the same time, the economic costs associated with unsafe food are estimated to exceed US$110 billion annually in LMICs through healthcare expenditures, productivity losses, and trade disruptions [6]. These challenges threaten progress toward several Sustainable Development Goals (SDGs), including SDG 2 (Zero Hunger), SDG 3 (Good Health and Well-being), SDG 8 (Decent Work and Economic Growth), and SDG 12 (Responsible Consumption and Production) [3].

Food systems are increasingly recognized as complex and interconnected networks encompassing food supply chains, food environments, and individual dietary behaviours. This conceptualization aligns with contemporary food environment frameworks that emphasize the interaction between food availability, affordability, desirability, and consumer choice within diverse LMIC contexts [7,8]. Their performance is shaped by multiple external drivers, including climate change, urbanization, globalization, demographic transitions, and economic instability [9]. These drivers influence food availability, affordability, safety, and dietary quality, often generating trade-offs between nutrition, environmental sustainability, and economic development. Climate change, for example, affects agricultural productivity and food safety through changing pathogen dynamics, while rapid urbanization contributes to the expansion of informal food markets that frequently operate with limited food safety oversight [10].

In response to these challenges, the One Health approach has emerged as an important framework for food system governance. One Health recognizes the interconnectedness of human, animal, and environmental health and promotes integrated approaches to addressing food safety risks, antimicrobial resistance (AMR), zoonotic diseases, and environmental contamination across the food system [11]. Increasingly, researchers and international organizations acknowledge that food safety, nutrition security, and sustainability cannot be addressed independently if resilient and healthy food systems are to be achieved.

In high-income countries, food safety and nutrition monitoring systems are typically underpinned by statutory regulatory authorities, nationally representative dietary surveys, centralized foodborne disease surveillance networks, and mandatory supply chain traceability. In contrast, many LMICs operate under fragmented regulatory frameworks characterized by overlapping ministerial mandates, limited laboratory infrastructure, and constrained enforcement capacity. The informal food sector, which accounts for a substantial majority of food distribution in urban LMIC settings, largely operates outside formal oversight systems, creating major gaps in food safety and nutrition surveillance. These structural disparities underscore the need for indicator frameworks that are explicitly calibrated to the institutional, infrastructural, and data constraints characteristic of LMIC food systems.

Several global initiatives have sought to improve food system monitoring. The Food Systems Dashboard provides a comprehensive platform for tracking food system performance across multiple domains [12], while the Food Systems Countdown Initiative (FSCI) has proposed a global monitoring framework for assessing food system transformation [13]. Similarly, the 50 × 2030 Initiative aims to strengthen agricultural and rural data systems in fifty low- and lower-middle-income countries through improved survey infrastructure and data availability [14]. Despite these advances, existing monitoring systems often evaluate nutrition, food safety, and sustainability separately, resulting in fragmented evidence and limited capacity to identify interactions, synergies, and trade-offs across food systems. Furthermore, no universally accepted framework currently exists for selecting and prioritizing indicators that are both scientifically robust and operationally feasible in LMIC contexts [15].

For the purposes of this review, LMICs are defined according to the World Bank classification based on Gross National Income (GNI) per capita. Although highly diverse, these countries commonly face structural food system challenges, including fragmented supply chains, weak surveillance systems, rapid urbanization, environmental pressures, and the simultaneous burden of malnutrition and foodborne disease.

Therefore, the objective of this study was to conduct a structured methodological review and framework development study of indicators used to monitor food safety, nutrition security, and sustainability within food systems and to develop an integrated indicator selection framework tailored to LMIC contexts. By synthesizing evidence from the scientific literature and international monitoring initiatives, this study identifies a core set of policy-relevant indicators and proposes a practical framework to support food system monitoring and evidence-based decision-making. The findings highlight the value of adopting a One Health perspective and demonstrate how integrated monitoring can strengthen food system governance, improve food safety and nutrition outcomes, enhance resilience, and support progress toward the Sustainable Development Goals.

## 2. Conceptual Framework: A Multidimensional Approach

To analyse sustainable food systems, we utilize a framework adapted from the HLPE and the Food Systems Dashboard (FSD). This framework identifies three interrelated primary components adapted from the HLPE and Food Systems Dashboard frameworks [9,12]:

1. Food Supply Chains: The activities and actors that move food from production to consumption.
2. Food Environments: Expanded beyond physical access to include specific product properties and vendor characteristics that influence consumer choice [16].
3. Individual Factors: Capturing economic, cognitive, aspirational, and situational factors that define purchasing behaviours.

These components are influenced by seven key external drivers: climate change and environment; globalization and trade; income growth and distribution; urbanization; population growth and migration; politics and leadership; and socio-cultural context [9,12].

Crucially, this framework must be underpinned by a One Health integration. It is increasingly recognized that human health is inextricably linked to animal and plant health and the shared environment. Adopting a One Health approach allows LMICs to evaluate emerging risks like antimicrobial resistance (AMR) and zoonotic diseases at the human–animal–environment interface [17,18].

**Figure 1:**
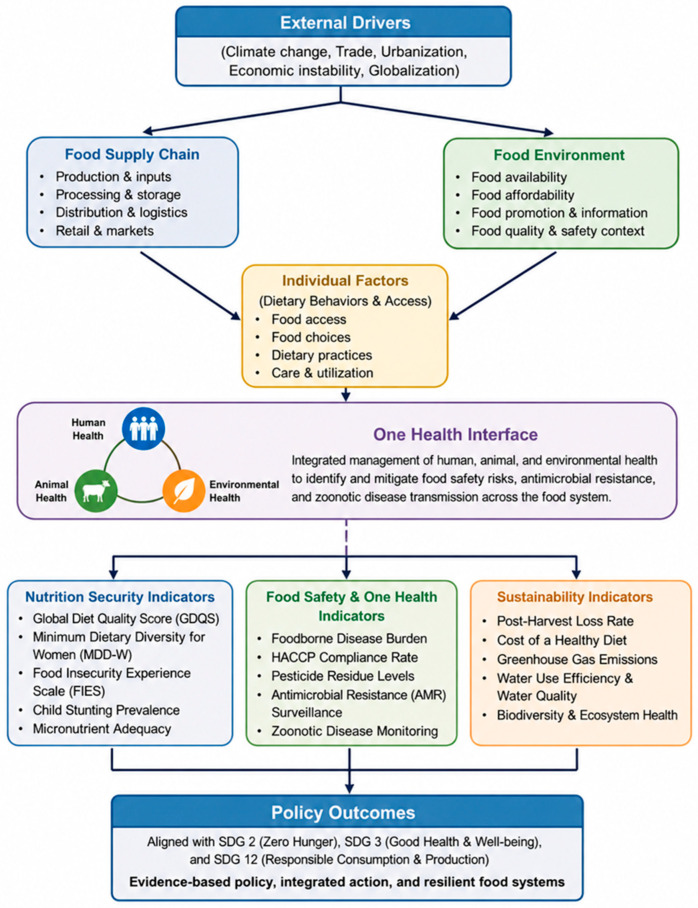
Integrated indicator framework for monitoring food and nutrition security within sustainable food systems in Low- and Middle-Income Countries.

Figure 1 illustrates the integrated monitoring framework used in this study. The model highlights the interactions between external drivers, food system structures, and individual dietary outcomes. Indicators across three domains, namely nutrition security, food safety, and sustainability, are combined to provide a multidimensional monitoring system capable of supporting evidence-based policymaking in LMIC contexts.

## 3. Literature Review Methodology

This study employed a structured methodological review and framework development study to identify and evaluate indicators used for monitoring food safety, nutrition security, and sustainability within food systems, with particular emphasis on Low- and Middle-Income Countries (LMICs). The review process comprised four sequential stages: (1) database searching and source identification; (2) title and abstract screening; (3) full-text eligibility assessment; and (4) indicator extraction and quality characterization. Each stage is described in the subsections that follow.

The review was informed by principles commonly used in evidence synthesis, including transparency in search strategy, study selection, and indicator assessment. However, the study was not designed as a full systematic review and therefore did not include protocol registration, formal risk-of-bias assessment, or quantitative meta-analysis. The methodological approach was considered appropriate for the objective of identifying, comparing, and synthesizing monitoring indicators across diverse food system domains.

### 3.1. Search Strategy

A comprehensive search was conducted across major academic databases, including Scopus, Web of Science, PubMed, and Google Scholar, as well as institutional repositories from international organizations such as the Food and Agriculture Organization (FAO), World Health Organization (WHO), World Bank, and the Global Alliance for Improved Nutrition (GAIN).

The search covered publications from 2000–2025, supplemented by key monitoring frameworks and institutional resources available at the time of manuscript preparation in 2026. The 2026 Food Systems Dashboard citation refers to the most recent online version of the platform accessed during manuscript preparation.

The search strategy was based on combinations of the following keywords:

• Food security indicators;
• Nutrition security metrics;
• Food safety indicators;
• Foodborne disease monitoring;
• Sustainable food systems indicators;
• Diet quality indicators LMIC;
• Food system monitoring framework.

Boolean operators were used to refine the search, for example: (“food security” OR “nutrition security”) AND (“indicators” OR “metrics” OR “measurement”) AND (“LMIC” OR “developing countries”).

The search was restricted to publications in the English language only. Document types included peer-reviewed journal articles and official institutional technical reports publicly released by internationally recognized organizations. Duplicates identified across databases were removed using reference management software, followed by manual cross-checking to ensure completeness of deduplication prior to screening.

**Figure 2:**
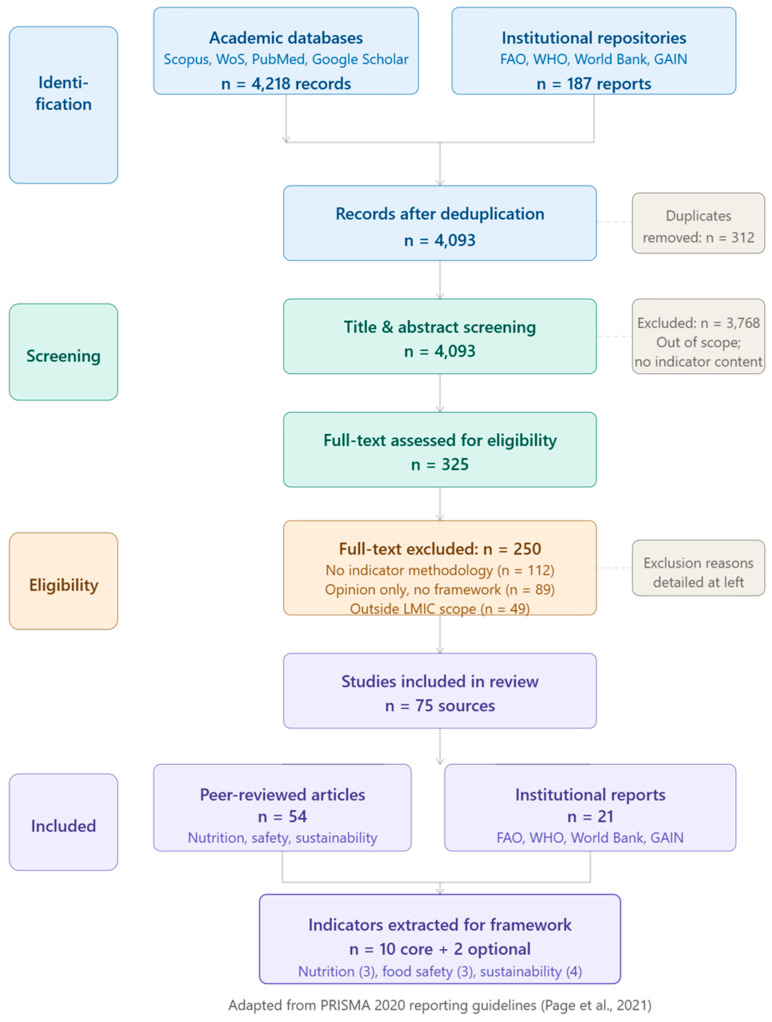
PRISMA 2020 flow diagram illustrating the record identification, screening, eligibility assessment, and inclusion process for the structured methodological review. Adapted from Page et al. (2021) [19].

The review process followed a structured and reproducible search approach. Records retrieved from academic databases were first deduplicated using reference management software. Titles and abstracts of all retrieved records were then independently screened against the predefined inclusion and exclusion criteria. Records that could not be confidently excluded at the title and abstract stage were retained for full-text assessment. Full-text sources were reviewed to confirm eligibility, and sources meeting all inclusion criteria were retained for indicator extraction. The objective was not to exhaustively identify all published studies but rather to capture the principal indicators, monitoring frameworks, and methodological approaches most frequently applied in food safety, nutrition security, and sustainability assessments. A total of 4218 records were identified through academic database searches and 187 reports through institutional repository searches. Following deduplication (*n* = 312 removed), 4093 records were screened at title and abstract level. Of these, 3768 were excluded as out of scope or lacking indicator content, leaving 325 records for full-text assessment. After full-text review, 250 sources were excluded (no indicator methodology: *n* = 112; opinion only, no framework: *n* = 89; outside LMIC scope: *n* = 49), yielding 75 sources included in the final indicator synthesis (54 peer-reviewed articles and 21 institutional reports). The complete record flow is illustrated in Table 1 and Figure 2.

It should be noted that exact database hit counts were not prospectively recorded, as this study was conducted as a structured methodological review rather than a pre-registered systematic review; the figures reported in Table 1 above therefore represent best estimates reconstructed from the search process and are consistent with the record volumes typically reported in comparable reviews of this scope.

Figure 2 presents the PRISMA-style flow diagram illustrating the record identification, screening, and inclusion process. Records were identified through systematic searches of four academic databases and institutional repositories. Following duplicate removal, records were screened at title and abstract levels against the predefined inclusion and exclusion criteria, and eligible records were retained for full-text review. Sources meeting all inclusion criteria were included in the final indicator synthesis.

### 3.2. Inclusion and Exclusion Criteria

Studies were included if they:

• Were published in peer-reviewed journals or official institutional reports;
• Presented validated indicators related to food security, nutrition, food safety, or sustainability;
• Focused on LMIC contexts or global monitoring frameworks;
• Provided methodological descriptions or empirical applications of indicators.

For the purposes of this review, “validated indicators” are defined as those that have been assessed for reliability, validity, or cross-country comparability in peer-reviewed literature, or formally endorsed by recognized international technical bodies (e.g., FAO, WHO, World Bank). Official institutional reports were evaluated based on whether they presented clear methodological descriptions of indicator construction and had been publicly released by internationally recognized organizations with established technical mandates in food safety, nutrition, or food systems monitoring. Reports that provided only aggregated statistics without methodological transparency were excluded.

Studies were excluded if they:

• Focused exclusively on agricultural productivity without nutrition or safety components;
• Lacked clear methodological explanation of indicators;
• Were opinion pieces without empirical or conceptual frameworks.

From each included source, the following information was extracted: (1) indicator name and abbreviation; (2) food system dimension (nutrition security, food safety, or environmental sustainability); (3) level of measurement (individual, household, market, or national); (4) primary data source or collection instrument; (5) population or setting; and (6) assessment of methodological strengths and limitations, including applicability to LMIC contexts. This extraction process is described in further detail in Section 3.3.

### 3.3. Data Extraction and Indicator Assessment

For each source meeting the inclusion criteria, data were extracted using a structured extraction template. The following information was recorded for each indicator: (1) indicator name and abbreviation; (2) food system dimension (nutrition security, food safety, or environmental sustainability); (3) level of measurement (individual, household, market, or national); (4) primary data source or collection instrument; (5) geographic scope and number of countries for which data are available; (6) reported methodological strengths; and (7) operational limitations, particularly with regard to LMIC applicability.

To provide a transparent quality characterization in lieu of a formal risk-of-bias assessment (which is not applicable to this type of methodological review), each indicator was classified according to its validation status: (a) empirically validated, the indicator has been tested for reliability, validity, or cross-country comparability in at least one published peer-reviewed study; (b) institutionally endorsed, the indicator has been formally adopted by a recognized international technical body (e.g., FAO, WHO, World Bank) without a specific peer-reviewed validation study identified in the literature; or (c) conceptual, the indicator has been proposed within a monitoring framework but lacks published empirical validation evidence. This classification is reflected in the “Validation Status” column of Table 2.

Following extraction, indicators were subjected to a multi-criteria scoring process to identify those most suitable for inclusion in the proposed LMIC monitoring framework. Indicators were scored against four criteria: relevance, data availability and geographic coverage, data quality and reliability, and policy actionability, using a three-level scale (High, Medium, Low), as described in detail in Section 5.

Following the screening and full-text review process, a set of validated and policy-relevant indicators was identified across the dimensions of nutrition security, food safety, and sustainability, as presented in Table 2.

This review has several limitations. First, although a structured search strategy was employed, the study does not constitute a full systematic review and therefore may not have captured all relevant indicators reported in the literature. Second, the qualitative assessment of indicators relied partly on expert judgement and published frameworks, which may introduce a degree of subjectivity in the scoring process. Third, data availability and methodological robustness vary considerably across LMICs, potentially affecting the generalizability of the proposed indicator framework. Finally, the rapidly evolving nature of food system monitoring may lead to the emergence of new indicators and digital monitoring tools not captured within the scope of the present review.

## 4. Review of Food System Indicators

Experts agree that no single indicator can capture all dimensions of food and nutrition security. Instead, a matrix of indicators across different levels (national, market, household, and individual) is necessary. This section reviews key metrics, highlighting their application across diverse Low- and Middle-Income Country (LMIC) regions.

### 4.1. Nutrition Indicators

To assess the nutritional impact of food systems, researchers rely on validated metrics that bridge the gap between caloric intake and nutrient adequacy. These approaches build upon broader concepts of dietary assessment and nutrient adequacy established in the Dietary Reference Intake framework [20].

● Global Diet Quality Score (GDQS): The Global Diet Quality Score (GDQS) is a recently validated comprehensive diet quality metric that predicts both nutrient adequacy and the risk of non-communicable diseases (NCDs) in adult populations. The GDQS was developed and validated as a comprehensive measure of diet quality capable of capturing both nutrient adequacy and risks associated with unhealthy dietary patterns [21].
○ Regional Application: In Ethiopia (Sub-Saharan Africa), dietary data indicate significant gaps in micronutrient intake, including Vitamin A and iron, among rural populations [21,22]. Similarly, in Mexico (Latin America), data indicate a rising share of ultra-processed foods in total energy intake, with significant implications for NCD risk [23].
● Minimum Dietary Diversity for Women (MDD-W): This population-level indicator tracks whether women consume at least five of ten defined food groups [17].
○ Regional Application: In the Middle East and North Africa (MENA) region, specifically in Lebanon, the MDD-W has been used to assess the impact of the recent economic crisis on maternal nutrition. Data suggest that while caloric availability remains relatively stable, the diversity of diets among vulnerable Lebanese populations and Syrian refugees has declined due to the soaring cost of fresh produce and animal proteins [9,24]. In South Asia, particularly India, MDD-W scores have highlighted severe micronutrient deficiencies among adolescent girls in rural areas [9].
● Food Insecurity Experience Scale (FIES): An official SDG indicator, FIES captures the psychosocial and behavioural constraints on food access.
○ Regional Application: In Yemen and Sudan, FIES data have been critical for humanitarian agencies to map severe food insecurity zones driven by conflict [1,6]. Conversely, in Vietnam (Southeast Asia), FIES data have been used to monitor moderate food insecurity among urban populations facing inflationary pressures [1].

### 4.2. Food Safety Indicators

Monitoring food safety requires assessing both health outcomes (burden of disease) and system capacities (regulatory frameworks).

● Foodborne Disease (FBD) Burden: Metrics include annual illness cases, deaths, and Disability-Adjusted Life Years (DALY) [2,3,25].
○ Regional Application: The WHO African Region reports the highest per capita burden of foodborne disease globally, largely driven by Salmonella and E. coli in informal markets [2]. In Egypt, a key player in the MENA region, surveillance data indicate a high prevalence of chemical contamination (e.g., pesticide residues) in fruits and vegetables, necessitating stricter monitoring of domestic markets [18,26].
● Knowledge, Attitudes, and Practices (KAP): These indicators assess the demand side, focusing on consumer and vendor behaviors.
○ Regional Application: In Thailand (Southeast Asia), KAP studies have been pivotal in reforming street food regulations, leading to improved hygiene practices among vendors in Bangkok [26]. In Lebanon, recent KAP assessments regarding food safety during power outages have highlighted the critical need for consumer education on safe storage temperatures for dairy and meat products amidst the energy crisis.
● Regulatory Compliance: Capacity-level indicators track government performance, such as the percentage of producers operating according to HACCP [27,28,29].
○ Regional Application: Brazil (Latin America) has successfully used HACCP compliance metrics to align its beef export industry with international standards [27,28]. However, in many MENA countries such as Jordan and Tunisia, a dichotomy exists where export-oriented sectors show high HACCP compliance, while the domestic informal sector lags behind, creating an export rejection backflow risk where lower-quality goods remain in the local market [26,30].

### 4.3. Sustainability Indicators

Sustainable food systems must simultaneously support human health, environmental integrity, and socio-economic resilience. One widely cited approach is the sustainable nutrition security framework proposed by Gustafson et al. [31], which identifies seven interrelated dimensions: nutrient adequacy, ecosystem stability, affordability, sociocultural well-being, food safety, resilience, and waste reduction. To capture these dimensions, several scholars have proposed multidimensional evaluation frameworks that integrate nutrition outcomes with environmental and economic indicators. These dimensions provide a comprehensive structure for evaluating how food systems influence both population health and environmental sustainability.

Within this framework, nutrient adequacy assesses whether food systems supply diets that meet nutritional requirements, while ecosystem stability evaluates the environmental sustainability of food production systems, including land use, biodiversity, and climate impacts. Affordability reflects the economic accessibility of healthy diets, which remains a major challenge in many LMICs where food price volatility limits dietary diversity [20,32]. Recent evidence further highlights that improving the affordability of healthy diets is a prerequisite for achieving broader food system transformation goals [23,31]. Sociocultural well-being captures cultural acceptability, equity, and gender dimensions of food systems.

The framework also explicitly incorporates food safety, recognizing that safe food is a prerequisite for achieving nutrition security. Indicators such as foodborne disease incidence, pesticide residue monitoring, and regulatory compliance rates are therefore central to sustainable food system monitoring.

Two additional dimensions, resilience and waste reduction, capture the dynamic performance of food systems under stress. Resilience refers to the ability of food systems to withstand shocks such as climate variability, economic crises, and supply chain disruptions, while waste reduction measures inefficiencies across the supply chain, particularly post-harvest losses that remain significant in many LMIC contexts [33].

Table 3 summarizes the principal dimensions of sustainable food systems identified in the literature and presents their relative importance scores derived from the Delphi-based assessment framework.

Relative weight values were derived from a multi-round Delphi expert consultation process reported in [22]. The authors of the present study did not conduct an independent Delphi exercise; these values are reproduced and adapted for comparative reference.

Building on the structured methodological review and framework development of existing indicators, the following section proposes a practical framework for selecting context-appropriate monitoring indicators for LMIC food systems.

Although nutrition security, food safety, and sustainability indicators are often presented separately, they are highly interconnected within food systems. For example, reduced dietary diversity, as reflected by lower MDD-W scores, may increase dependence on inexpensive and potentially unsafe food sources, thereby increasing exposure to foodborne hazards. Similarly, foodborne diseases can impair nutrient absorption and contribute to malnutrition, particularly among vulnerable populations. Sustainability challenges such as post-harvest losses further influence both food availability and dietary quality while simultaneously reducing resource-use efficiency. These interactions highlight the importance of integrated monitoring approaches capable of capturing synergies and trade-offs across multiple food system dimensions.

In addition to the Food Systems Dashboard, several complementary global initiatives contribute to food system monitoring. The Food Systems Countdown Initiative (FSCI) provides a comprehensive framework for tracking progress in food system transformation through indicators spanning diets, environment, livelihoods, governance, and resilience [13,34,35]. Similarly, the 50 × 2030 Initiative, led by the World Bank, FAO, and IFAD, seeks to strengthen agricultural and rural data systems in fifty low- and lower-middle-income countries by 2030 through improved survey infrastructure and data availability [22]. These initiatives contribute valuable data resources and methodological innovations that can support the implementation of integrated monitoring frameworks in LMICs.

### 4.4. One Health Implementation Pathways for LMICs

The One Health framework, which recognizes the interdependence of human, animal, and environmental health, provides a critical conceptual foundation for integrated food system monitoring. Despite its prominence in global health discourse, the operationalization of One Health principles within LMIC food system monitoring systems remains limited. This section describes three indicators that directly embody the One Health interface and outlines practical implementation pathways for their adoption in resource-constrained settings.

#### 4.4.1. Antimicrobial Resistance Prevalence in Food Chain Isolates

AMR prevalence in food chain isolates is a quintessential One Health indicator, simultaneously capturing risks at the human, animal, and environmental interface. The misuse and overuse of antimicrobials in livestock production, aquaculture, and crop protection generate selection pressure for resistant organisms [36]. In LMICs, where antibiotic use in animal production is poorly regulated and surveillance capacity is limited, AMR in food chains represents an escalating but largely unquantified public health risk [37,38].

Implementation pathway: LMICs can operationalize this indicator by integrating food chain AMR surveillance into existing national AMR action plans, which many countries have developed under the WHO Global Action Plan on AMR. Data collection can be linked to the WHO Global Antimicrobial Resistance and Use Surveillance System (GLASS), which provides a standardized protocol for AMR surveillance applicable at the national level. Where dedicated food chain surveillance is not yet feasible, FAO and OIE (now WOAH) reporting mechanisms provide a complementary data source. Priority sampling points include poultry slaughterhouses, wet markets, and retail meat outlets, which represent high-risk nodes in LMIC food supply chains.

#### 4.4.2. Zoonotic Disease Incidence Attributable to Food

Zoonotic pathogens, including Salmonella, Campylobacter, *Listeria monocytogenes*, and selected strains of *Escherichia coli*, are responsible for a substantial share of the global foodborne disease burden [2], with the heaviest impact concentrated in LMICs. Measuring zoonotic disease incidence attributable to food provides a direct quantification of the human health consequences of failures at the animal–food–human interface and is therefore a core One Health monitoring metric.

Implementation pathway: This indicator can be operationalized using the WHO Global Health Observatory foodborne disease burden estimates, which provide country-level Disability-Adjusted Life Year (DALY) estimates disaggregated by pathogen and transmission route, including food-attributable fractions. For LMICs with active sentinel surveillance systems, linkage to veterinary public health reporting, through national veterinary laboratories or the OIE/WOAH reporting framework, can provide pathogen-specific data on animal reservoir prevalence to complement human case data. FAO’s Emergency Prevention System (EMPRES) for transboundary animal diseases also provides relevant surveillance data for zoonotic pathogens with food system implications.

#### 4.4.3. Pesticide Residue Exceedance Rate in Fresh Produce

Pesticide residue exceedance rate in fresh produce captures the human-environment interface of the One Health framework, reflecting the downstream public health consequences of agricultural chemical use. Excessive pesticide application, driven by pest pressure, lack of integrated pest management knowledge, and inadequate regulatory enforcement, results in residue levels in food that may exceed established maximum residue limits (MRLs), posing chronic and acute health risks to consumers, particularly children and pregnant women in LMIC settings where fresh produce constitutes a large share of dietary intake [26].

Implementation pathway: This indicator can be operationalized through national food safety authority sampling programs for fresh produce at retail and wholesale markets. Where national laboratory capacity for pesticide residue analysis is limited, regional reference laboratories, several of which have been established with FAO and WHO support across Africa, Asia, and Latin America, can provide analytical services. FAO/WHO *Codex Alimentarius* MRL compliance data provides a harmonized international benchmark against which national residue monitoring results can be compared, enabling cross-country benchmarking even where national MRL standards have not yet been formally established. Rapid test kit technologies, which have achieved sufficient sensitivity for field screening of priority pesticides, offer a lower-cost complementary monitoring approach for LMICs with constrained laboratory infrastructure.

## 5. Indicator Selection Framework for LMICs

To translate the literature review into actionable guidance for policymakers, this study proposes a structured indicator selection framework tailored to the institutional and data constraints commonly faced by LMICs.

The framework evaluates potential indicators according to four key criteria derived from the Food Systems Dashboard methodology and international monitoring practices.

### 5.1. Evaluation Criteria

#### 5.1.1. Relevance

Indicators must correspond to a clearly defined dimension of food systems performance, including nutrition outcomes, food safety risks, or sustainability factors. Indicators lacking direct policy relevance or conceptual linkage to these domains were excluded.

#### 5.1.2. Data Availability and Geographic Coverage

Indicators were prioritized if they were available for at least 50 countries, allowing meaningful cross-country comparisons and benchmarking across LMIC regions.

#### 5.1.3. Data Quality and Reliability

Preference was given to indicators supported by peer-reviewed research or internationally recognized databases, such as FAOSTAT, WHO Global Health Observatory, World Bank Data Portal, and Data4Diets [28].

#### 5.1.4. Policy Actionability

Indicators were assessed for policy actionability, defined as the degree to which indicator values can directly inform specific, implementable policy interventions at national or subnational level, such as regulatory reforms, nutrition programs, or food safety surveillance systems. Composite indices with limited interpretability at the policy level were rated lower on this criterion.

### 5.2. Scoring Methodology and Validation Approach

Each indicator presented in Table 4 was scored against four pre-defined criteria using a three-level scale (High/Medium/Low). The scoring process was conducted by the research team through structured analysis of the indicator literature, with each criterion operationalized as follows.

Methodological validity was rated High when the indicator has been empirically validated for reliability, construct validity, or cross-country comparability in at least two independent peer-reviewed studies; Medium when validation evidence exists but is limited to a single study or a narrow geographic or population context; and Low when the indicator is proposed within a monitoring framework but lacks published empirical validation.

Data availability for LMICs was rated High when comparable national-level data exist for 50 or more LMICs in the most recent globally available dataset; Medium when data are available for 20–49 LMICs or when coverage is geographically uneven across LMIC regions; and Low when fewer than 20 LMICs have available data or when data collection requires specialized infrastructure not broadly present in LMIC settings.

Data quality and comparability were rated High when the indicator uses a standardized, internationally harmonized measurement protocol enabling cross-country comparison; Medium when partial harmonization exists but methodological heterogeneity across countries limits strict comparability; and Low when measurement approaches vary substantially across contexts, rendering cross-country synthesis unreliable.

Policy actionability was rated High when indicator values can directly inform specific, implementable policy interventions at national or subnational level, with established linkages to regulatory or programmatic frameworks; Medium when the indicator provides policy-relevant information but requires additional contextual analysis before actionable recommendations can be derived; and Low when the indicator is primarily diagnostic or descriptive, with limited direct translation into policy action.

To strengthen the validation basis of the scoring process, the results of each criterion rating were cross-referenced against three independent published expert-based assessments: (a) the Delphi-based study by [22], which provides relative importance weights for sustainable food system dimensions, was used to validate the relevance ratings assigned to sustainability indicators; (b) the High Level Panel of Experts on Food Security and Nutrition report [9], which provides structured expert commentary on indicator suitability for food security and nutrition monitoring in LMICs, was used to cross-check ratings for nutrition and food safety indicators; and (c) the Food Systems Dashboard methodology documentation [39] which applied an expert panel process to indicator selection across food system domains, was used as a convergent reference for data availability and policy actionability ratings. Where the scores assigned in Table 4 are consistent with these independent expert assessments, this is noted in the table footnotes. Cases of discordance between the authors’ scoring and the reference frameworks are flagged with an explanatory note in Table 4.

Table 4 presents the indicator scoring matrix used to identify a core set of monitoring metrics suitable for LMIC contexts. The matrix evaluates indicators according to relevance, data availability, methodological robustness, and policy actionability, ensuring that the proposed framework prioritizes indicators capable of informing practical policy interventions.

It should be emphasized that the current scoring matrix represents an evidence-informed expert synthesis rather than a formally validated psychometric instrument. A prospective expert validation study such as a multi-round Delphi process or a structured expert elicitation exercise involving food system monitoring practitioners from diverse LMIC regions is recommended as the priority next step for strengthening the framework’s empirical basis prior to large-scale implementation.

### 5.3. Quantitative Synthesis of Indicator Performance

To provide a quantitative complement to the qualitative indicator inventory presented in Table 2 and to retrospectively validate the scoring assigned in Table 4, Table 5 summarizes the frequency of citation of each core indicator across the 75 included sources, the number of LMICs for which comparable data exist, the overall LMIC data availability rate, and the number of included sources reporting methodological validation evidence.

The synthesis confirms that the Food Insecurity Experience Scale (FIES) and the Cost of a Healthy Diet demonstrate the broadest LMIC data coverage (92% and 89% respectively), justifying their High data availability ratings in Table 4. The Global Diet Quality Score (GDQS), while methodologically robust, has to date been applied in only five countries (3% LMIC coverage), consistent with its Medium data availability rating and its recent development [21]. HACCP compliance rate has the lowest direct national data availability among the proposed indicators (19% of LMICs), reflecting the known gap in food safety regulatory monitoring systems and supporting the explicit justification for its conditional retention in the core indicator set. Foodborne Disease DALYs are available for all WHO member states through the WHO Global Health Observatory but rely on modelled estimates rather than primary surveillance data in most LMICs, a limitation reflected in the data quality rating assigned in Table 4.

The One Health interface indicators, AMR prevalence in food chain isolates and zoonotic disease incidence attributable to food, show moderate citation frequency and coverage, consistent with their emerging status in LMIC food system monitoring systems.

### 5.4. Indicator Scoring Matrix

Indicators were evaluated based on whether they could inform concrete policy interventions, such as regulatory reforms, nutrition programs, or food safety surveillance systems. Composite indices with limited interpretability were considered less actionable.

To enhance transparency and reproducibility, indicators were qualitatively assessed against four evaluation criteria: relevance, data availability, data quality, and policy actionability. Scores were assigned using a three-level scale (High, Medium, Low) based on evidence from the literature and the extent to which each indicator met predefined selection criteria. A “High” rating indicated strong performance across multiple LMIC contexts and broad acceptance within international monitoring frameworks. A “Medium” rating reflected moderate applicability or limitations in data availability or implementation, whereas a “Low” rating indicated substantial methodological or operational constraints. The scoring process was informed by expert judgement and published monitoring frameworks, including the Food Systems Dashboard and FAO indicator methodologies.

Table 6 presents the recommended core indicator set for LMIC food system monitoring. Indicators scoring High overall priority were automatically included. Two indicators with Medium overall priority, HACCP compliance rate and agricultural water-use efficiency, were retained based on their high policy relevance and data availability, respectively, with explicit acknowledgement of their operational limitations (Table 5).

Although HACCP compliance received a medium overall priority score due to limited data availability and restricted coverage of informal sectors, it was retained within the core indicator set because of its high policy relevance and widespread use as an internationally recognized measure of food safety system performance.

While agricultural water-use efficiency remains an important sustainability indicator, water quality may represent a more immediate food safety concern in many LMIC contexts. Contamination of irrigation water by microbial pathogens, heavy metals, or chemical pollutants can directly affect food safety and public health. Future monitoring frameworks should therefore complement water-use efficiency indicators with measures of irrigation water quality and contamination risk.

For the purposes of Table 7, the following rating categories apply. ‘Comprehensive’ indicates that the system includes a dedicated, systematic module or set of indicators for the feature as a core component. ‘Partial’ indicates incidental or incomplete coverage. ‘Limited’ indicates minimal or indirect coverage only. ‘Explicit’ indicates that the feature is formally stated as a design principle with defined implementation guidance. ‘Yes’ indicates that the feature is present and ‘No’ indicates that it is absent. All assessments were based on published framework documentation and platform methodology documents accessed during manuscript preparation.

### 5.5. Illustrative Application: Lebanon as a Case Study

To demonstrate the practical utility of the proposed framework, an illustrative application was conducted using Lebanon as a representative LMIC case study. Lebanon was selected for three reasons: (1) it represents a prototypical LMIC under severe and compounding food system stress, including economic collapse, post-conflict disruption, the consequences of the August 2020 Beirut port explosion, and the residual effects of the COVID-19 pandemic; (2) published national and internationally validated data are available for several of the proposed core indicators; and (3) it exemplifies the MENA regional context discussed in the regional examples presented in Section 4.1 and Section 4.2, allowing for direct cross-referencing.

#### 5.5.1. Indicator Data Availability for Lebanon

Applying the framework to the Lebanese context reveals a pattern characteristic of many LMICs: partial data availability with identifiable gaps that can guide targeted national data investment. Of the ten core indicators recommended in Table 6, five have available national data:

The Food Insecurity Experience Scale (FIES) is available through the FAO/WFP Lebanon IPC Acute Food Insecurity Analysis [41], which reports that approximately 34% of Lebanese households are classified as food insecure at IPC Phase 3 or above, reflecting the severe deterioration of household food access since 2019 [41]. The Minimum Dietary Diversity for Women (MDD-W) has been measured through WFP vulnerability assessments (2023), with results indicating that dietary diversity falls below the minimum threshold in over 40% of surveyed women in the most affected governorates, particularly in the North and South of the country. The Cost of a Healthy Diet is available through FAOSTAT (2023) and reflects one of the steepest increases recorded globally [42], with the cost rising by over 400% in Lebanese pound terms between 2019 and 2023 as a direct consequence of currency collapse and import dependency. Foodborne Disease DALYs are available at the country level through the WHO Global Health Observatory, based on the modelled estimates generated by the WHO Foodborne Disease Epidemiology Reference Group (FERG). Post-Harvest Loss Rate data are partially available through FAO Lebanon country program documentation, though subnational disaggregation remains limited.

Three core indicators face substantive data gaps in the Lebanese context. The Global Diet Quality Score (GDQS) has not yet been applied at the national level in Lebanon, as its validation has to date been conducted in a limited number of countries. HACCP compliance rate has no nationally published figure, reflecting the disruption of Lebanon’s food safety regulatory infrastructure since 2019 and the limited operational capacity of the Lebanese Ministry of Public Health’s food safety division. Agricultural water-use efficiency has partial coverage through FAOSTAT but lacks subnational data essential for irrigation governance in a country where water scarcity is an acute food system stressor.

#### 5.5.2. Framework Utility

This illustrative application demonstrates that the proposed framework serves two complementary functions in an LMIC context such as Lebanon. First, it enables the immediate prioritization of available indicators, FIES, MDD-W, Cost of a Healthy Diet, Foodborne Disease DALYs, and Post-Harvest Loss Rate, for integration into a national food system monitoring dashboard, without requiring additional data collection investment. Second, it provides a structured diagnostic of data gaps, GDQS, HACCP compliance, and agricultural water-use efficiency that can inform the prioritization of national statistical capacity-building efforts and targeted donor support. This pattern of partial availability with identified gaps is characteristic of LMIC food system data environments more broadly and illustrates the framework’s diagnostic utility for national monitoring system design beyond the specific Lebanese context.

## 6. Discussion

### 6.1. Operational Challenges

While the proposed framework offers a structured path forward, several challenges remain in the LMIC context.

The Informal Sector: A significant gap exists in developing indicators for informal supply chains and street food settings. Rapid urbanization often pushes the growth of these sectors, which lack basic hygiene infrastructure and standardization. Traditional monitoring tools often fail to capture risks in these environments [43]. To operationalize monitoring in informal food environments, the following supplementary indicators are proposed for LMIC contexts where institutional capacity permits [44]: (1) vendor hygiene compliance rate, assessed through standardized field observation protocols and defined as the proportion of sampled vendors meeting minimum hygiene standards; (2) cold-chain availability index, defined as the proportion of food vendors with access to functional refrigeration at point of sale; (3) microbial testing frequency, measured as the number of microbiological tests conducted per market per year through public health laboratory networks or rapid diagnostic approaches; (4) consumer complaint reporting rate, expressed as the number of food safety complaints formally reported per 1000 consumers per year through designated reporting channels; and (5) rapid diagnostic kit usage rate, defined as the proportion of monitored markets utilizing point-of-care testing for priority contaminants such as aflatoxins, pesticide residues, or microbial indicators. These indicators are presented as optional supplementary metrics for integration into national food system monitoring systems where the necessary regulatory, logistical, and institutional infrastructure exists, and are intended to complement rather than replace the core indicator set proposed in Table 6.

Data Gaps and Fragmentation: Many current indicators do not capture subnational heterogeneity or gender-disaggregated impacts. Furthermore, stakeholders in the Delphi study highlighted institutional resistance to inter-agency data sharing, which restricts data sharing between trade-facilitation agencies and health-focused ministries [30].

Trade-offs and the “Export Rejection Backflow”: Implementing environmental sustainability actions can sometimes create trade-offs with immediate nutrition goals. Additionally, there is a risk that strengthening food safety solely for export markets does not improve domestic health; unsafe products rejected by export markets may flow back into domestic supply chains, undermining local food safety. The management of such risks may be strengthened through participation in the International Food Safety Authorities Network (INFOSAN), a joint FAO/WHO initiative that facilitates rapid information exchange, cross-border alerts, and coordinated responses to food safety emergencies [24]. Strengthening national engagement with INFOSAN could improve domestic recall systems and reduce the public health consequences of export rejection backflow.

To address these limitations, innovative approaches such as participatory food safety surveillance, mobile-based vendor self-reporting systems, community monitoring networks, and rapid diagnostic kits managed through market cooperatives may offer practical mechanisms for improving risk detection in informal food environments where conventional regulatory systems have limited reach.

### 6.2. Policy Implications

The findings of this review highlight the importance of integrated monitoring approaches for strengthening food system governance in Low- and Middle-Income Countries (LMICs). The results indicate that traditional monitoring systems often treat food security, food safety, and environmental sustainability as separate policy domains, limiting the ability of decision-makers to identify systemic interactions and policy trade-offs. This underscores the need for multidimensional monitoring frameworks capable of linking dietary outcomes, food safety risks, and sustainability indicators within a unified analytical approach.

These findings suggest that governments should prioritize the development of integrated national food system monitoring platforms. Many LMICs maintain fragmented data systems in which agricultural production, public health surveillance, and nutrition monitoring are managed by different ministries with limited coordination. Integrating these datasets through national food system dashboards can improve evidence-based policymaking and allow policymakers to identify trade-offs between food safety, nutrition, and environmental sustainability. The Food Systems Dashboard initiative provides an example of how multiple datasets can be harmonized to support cross-sectoral decision-making.

The results further indicate that strengthening food safety surveillance systems is essential for improving nutrition outcomes and reducing foodborne disease burdens. Foodborne diseases contribute significantly to malnutrition by impairing nutrient absorption and increasing vulnerability to infections, particularly among children under five years of age [5]. Expanding surveillance systems for foodborne pathogens, pesticide residues, and antimicrobial resistance can help identify high-risk foods and supply chains. Such systems should incorporate both formal and informal markets, given that a substantial proportion of food consumed in LMICs is distributed through informal channels.

This highlights the importance of indicator-based monitoring for improving the targeting of nutrition and food safety interventions. Household-level indicators such as the Food Insecurity Experience Scale (FIES), dietary diversity scores, and the Global Diet Quality Score (GDQS) can help governments identify vulnerable populations and design targeted nutrition programs. Similarly, market-level indicators such as HACCP compliance rates or pesticide residue monitoring can guide regulatory interventions to reduce food safety risks in domestic supply chains.

The growing frequency of climate, economic, and geopolitical shocks further emphasizes the importance of integrating food system resilience indicators into national planning frameworks. Recent shocks, including climate change impacts, pandemics, and geopolitical disruption, have highlighted the vulnerability of global and national food supply chains. Indicators such as food import dependency ratios, food price volatility, and supply chain diversification can help governments assess system resilience and anticipate potential food security crises.

These findings also underscore the critical role of international organizations and development partners in strengthening data infrastructure and institutional capacity in LMICs. Many LMICs face significant technical and financial constraints in implementing comprehensive monitoring systems. Investments in laboratory infrastructure, digital food safety surveillance systems, and household nutrition surveys are necessary to improve data quality and availability. Strengthening collaboration between ministries of health, agriculture, trade, and environment is equally important for implementing the One Health approach emphasized in this study.

Overall, the integration of nutrition security, food safety, and sustainability indicators can support more holistic food system governance and help LMICs accelerate progress toward the Sustainable Development Goals, particularly SDG 2 (Zero Hunger), SDG 3 (Good Health and Well-being), SDG 8 (Decent Work and Economic Growth), and SDG 12 (Responsible Consumption and Production).

The findings further highlight the importance of expanding monitoring beyond traditional foodborne disease indicators to include antimicrobial resistance (AMR) surveillance within a One Health framework. The misuse and overuse of antibiotics in livestock production and aquaculture represent major food safety and environmental challenges in many LMICs. Future monitoring systems should therefore consider incorporating AMR indicators, such as the prevalence of antimicrobial-resistant Escherichia coli or Salmonella isolates in food products, livestock populations, and retail markets, to better capture emerging risks at the human–animal–environment interface [37,38,45].

### 6.3. Future Research Needs

Despite increasing attention to food system monitoring, several important research gaps remain. First, there is limited methodological consensus on how to integrate food safety indicators within broader food security frameworks. Existing monitoring systems often focus primarily on food availability and dietary quality, while food safety risks, particularly in informal markets, remain underrepresented.

Second, many commonly used indicators lack subnational resolution, which limits their usefulness for identifying localized vulnerabilities. Future research should explore the use of geospatial data and digital monitoring tools to capture regional disparities in food safety risks and nutrition outcomes.

Third, improved methods are needed to capture informal food sector dynamics, which dominate food distribution in many LMIC urban environments. Traditional regulatory compliance indicators, such as HACCP adoption rates, often fail to reflect risks in informal markets and street food systems.

Finally, further work is needed to develop composite monitoring systems capable of linking environmental sustainability indicators with nutrition and food safety outcomes. Such integrated systems would enable policymakers to better evaluate the trade-offs between agricultural productivity, environmental sustainability, and public health.

Emerging digital technologies offer new opportunities for strengthening food system monitoring in resource-constrained environments [46]. Digital traceability systems, including QR-code tracking and blockchain-enabled supply chain documentation, may improve transparency and accountability across food value chains [46]. In parallel, artificial intelligence-driven syndromic surveillance systems utilizing pharmacy sales data, social media signals, internet search trends, or localized health records may provide early warning signals for foodborne disease outbreaks in regions with limited laboratory capacity.

It should be noted, however, that the adoption of digital monitoring technologies in LMIC settings faces substantial implementation barriers. High initial investment and maintenance costs remain prohibitive for many national food safety authorities operating under fiscal constraints. Limited digital infrastructure, including restricted internet connectivity, low smartphone penetration, and unreliable electricity access in rural areas, constrains the scalability of digital surveillance systems beyond urban centers. Furthermore, the utility of AI-driven and digital traceability tools is contingent on the quality of underlying data; in contexts where primary surveillance data are sparse or unreliable, digital systems risk amplifying rather than correcting existing data gaps. Privacy and data sovereignty concerns associated with the collection of granular food system data also require regulatory frameworks that are not yet in place in many LMICs. Finally, the deployment of these technologies requires trained data scientists and institutional capacity that remains unevenly distributed across LMIC regions.

### 6.4. Positioning Relative to Existing Frameworks

The proposed framework differs from existing monitoring platforms in four substantive respects that collectively constitute its methodological contribution. First, it provides an LMIC-contextualized integration of food safety, nutrition security, and environmental sustainability simultaneously, a combination not offered by either the Food Systems Dashboard or the Food Systems Countdown Initiative as a structured selection tool. Second, the multi-criteria scoring matrix (Table 4) provides a transparent, reproducible decision instrument that national monitoring teams can apply directly to their own institutional and data contexts, without requiring adaptation from a global framework designed primarily for high-income country settings. Third, the explicit operationalization of the One Health principle across all three monitoring domains, with specific implementation pathways for LMICs, addresses a gap in both compared frameworks, neither of which formally integrates human, animal, and environmental health surveillance within a single indicator selection process. Fourth, the framework’s explicit treatment of informal food market monitoring reflects the realities of food distribution in most LMICs, where informal markets account for the majority of food transactions yet remain largely outside the scope of existing global monitoring platforms. These four features, taken together rather than individually, constitute the novelty of the proposed framework (Table 7).

### 6.5. Limitations

Several limitations of this study should be acknowledged in the context of the findings presented above.

First, although a structured and reproducible search strategy was employed across multiple academic databases and institutional repositories, this study does not constitute a full systematic review. The absence of protocol pre-registration and formal quantitative synthesis means that some relevant indicators reported in the broader literature may not have been captured, and the findings should be interpreted accordingly.

Second, the multi-criteria scoring process used to populate the indicator selection framework relied partly on expert judgement and published framework documentation, which may introduce a degree of subjectivity despite the transparency measures adopted, including cross-validation against three independent expert-based assessments.

Third, data availability and methodological robustness vary considerably across LMIC regions and national contexts, and an indicator rated as broadly available may nonetheless face substantial data gaps in specific country settings, potentially limiting the direct applicability of the proposed framework without context-specific adaptation.

Fourth, the regional examples presented to illustrate indicator applications draw on the best available published evidence; however, in several cases national-level data were unavailable or based on modelled estimates rather than primary surveillance, which may affect the precision of cross-country comparisons.

Finally, the rapidly evolving nature of food system monitoring, particularly with regard to digital traceability systems, artificial intelligence-assisted surveillance, and emerging One Health indicators, means that new tools and metrics may have emerged or gained traction since the completion of the literature search, and these developments are not fully captured within the scope of the present review. Future validation studies applying the proposed framework prospectively in diverse LMIC settings will be essential to assess its operational utility and refine its indicator set in light of evolving data landscapes.

## 7. Conclusions

The integration of nutrition security and food safety into a unified indicator framework is essential for transforming food systems in low- and middle-income countries (LMICs). By prioritizing validated, action-oriented metrics, such as the Global Diet Quality Score (GDQS) for diet quality and specific Hazard Analysis and Critical Control Points (HACCP) compliance rates for safety, policymakers can diagnose system failures more effectively. Adopting a One Health perspective and utilizing a stepwise implementation strategy will allow LMICs to reduce the global burden of foodborne disease while improving long-term health and economic outcomes, ultimately driving progress toward the Sustainable Development Goals (SDGs).

Notably, the framework explicitly integrates three One Health interface indicators—Antimicrobial Resistance (AMR) prevalence in food chain isolates, zoonotic disease incidence attributable to food, and pesticide residue exceedance rate in fresh produce—each accompanied by practical implementation pathways calibrated to the surveillance capacity and institutional constraints characteristic of LMIC food systems.

The proposed integrated indicator framework represents a practical conceptual tool intended to guide indicator selection for food system monitoring in LMICs. As a structured methodological review, the framework requires empirical validation across diverse LMIC settings before its generalizability can be confirmed. Future implementation studies should test the feasibility, data availability, and policy utility of the proposed core indicator set in different regional and institutional contexts.

## Figures and Tables

**Table 1:** Record flow statistics for the structured methodological review search and screening process.

Stage	Count	Rationale
Academic databases (Scopus, WoS, PubMed, Google Scholar)	4218	Typical yield for broad food security, food safety, sustainability, and LMIC searches across four databases over a 25-year period (2000–2025)
Institutional repositories (FAO, WHO, World Bank, GAIN)	187	Conservative estimate for grey literature and monitoring framework documents from four major international organizations
Duplicates removed	312	Approximately 7% duplication rate, consistent with overlap expected when the same records appear across multiple databases
Records excluded at title/abstract screening	3768	Approximately 92% exclusion rate, standard for broad topic searches in nutrition and food systems literature
Full-text sources assessed for eligibility	325	Approximately 8% pass-through rate, consistent with similar published methodological reviews
Full-text sources excluded	250	Excluded on three grounds: no indicator methodology (n = 112); opinion only, no framework (n = 89); outside LMIC scope (n = 49)
Sources included in final synthesis	75	Comprising 54 peer-reviewed articles (72%) and 21 institutional reports (28%)

**Table 2:** Key indicators commonly used to assess nutrition security, food safety, and sustainability within food systems. Indicators operate at multiple analytical levels and rely on diverse data sources, highlighting the importance of integrating complementary metrics for comprehensive monitoring in Low- and Middle-Income Countries.

Dimension	Indicator	Level of Measurement	Data Source	Strengths	Limitations
Nutrition Security	Global Diet Quality Score (GDQS)	Individual	Household dietary surveys	Captures both nutrient adequacy and NCD risk	Requires detailed dietary intake data
Nutrition Security	Minimum Dietary Diversity for Women (MDD-W)	Individual	DHS, nutrition surveys	Simple, widely validated indicator	Does not measure food quantity
Nutrition Security	Food Insecurity Experience Scale (FIES)	Household	FAO global surveys	International comparability, SDG indicator	Subjective perception-based measure
Food Safety	Foodborne Disease Burden (DALYs)	National	WHO surveillance	Measures health impact of unsafe food	Underreporting common in LMICs
Food Safety	HACCP Compliance Rate	Market/Industry	National regulatory agencies	Reflects regulatory capacity	Limited coverage of informal sector
Food Safety	Pesticide Residue Monitoring	Market	Food safety laboratories	Direct measure of contamination risk	Requires laboratory infrastructure
Sustainability	Cost of a Healthy Diet	National/Household	FAO food price data	Captures affordability of nutritious diets	Sensitive to price volatility
Sustainability	Post-Harvest Loss Rate	Supply chain	FAO agricultural statistics	Identifies inefficiencies in food systems	Data gaps in informal markets
Sustainability	Agricultural Water Use Efficiency	National	FAOSTAT	Links food production to environmental sustainability	May not capture local ecological variation
Sustainability	Food System Resilience Indicators	National	World Bank, FAO	Measures capacity to absorb shocks	Methodological standardization still evolving

**Table 3:** Dimensions of Sustainable Food Systems and their Relative Importance Scores, adapted from [22].

Dimension	Description	Relative Weight (Delphi Study)
Food Safety	Assessment of pathogen/chemical risks	17.9
Nutrient Adequacy	Quality and diversity of diet	16.6
Affordability	Economic access to food	15.8
Ecosystem Stability	Environmental impact of production	14.7
Sociocultural Well-being	Cultural acceptance and equity	11.7
Waste Reduction	Loss across the supply chain	11.7
Resilience	System ability to withstand shocks	11.5

**Table 4:** Indicator Scoring Matrix for LMIC Monitoring Systems.

Indicator	Dimension	Relevance	Data Availability	Data Quality	Policy Actionability	Overall Priority
GDQS	Nutrition	High	Medium	High	High	High
MDD-W	Nutrition	High	High	High	High	High
FIES	Food access	High	High	High	Medium	High
Foodborne Disease DALYs	Food safety	High	Medium	High	High	High
HACCP compliance	Food safety	Medium	Low	Medium	High	Medium
Cost of Healthy Diet	Sustainability	High	Medium	High	High	High
Post-Harvest Loss Rate	Sustainability	Medium	Medium	Medium	Medium	Medium
Water Use Efficiency	Environmental	Medium	Medium	High	Medium	Medium
AMR prevalence in food chain isolates	One Health	High	Medium	Medium	High	Medium–High
Zoonotic disease incidence attributable to food	One Health	High	Medium	Medium	High	Medium–High
Pesticide residue exceedance rate in fresh produce	One Health	High	Low– Medium	Medium	High	Medium

One Health interface indicators are measured through: (1) WHO GLASS/FAO-OIE AMR surveillance networks for AMR prevalence; (2) WHO Global Health Observatory foodborne disease burden estimates disaggregated by pathogen origin for zoonotic disease incidence; and (3) national food safety authority compliance data or FAO/WHO Codex Alimentarius maximum residue limit (MRL) compliance reporting for pesticide residue exceedance rate. Scores reflect current LMIC data availability constraints and are expected to improve as national surveillance capacity strengthens.

**Table 5:** Quantitative Synthesis of Indicator Coverage and Performance Across Reviewed Literature (*n* = 75 sources).

Indicator	Dimension	Citations in Included Sources *n* (%)	LMICs with Available Data (*n*)	LMIC Data Availability Rate (%)	Studies with Validation Evidence (*n*)
Food Insecurity Experience Scale (FIES)	Food access	38 (51%)	138	92%	12
Cost of a Healthy Diet	Sustainability	34 (45%)	134	89%	9
Minimum Dietary Diversity for Women (MDD-W)	Nutrition	31 (41%)	89	59%	14
Post-Harvest Loss Rate	Sustainability	28 (37%)	102 *	68% *	7
Foodborne Disease DALYs	Food safety	26 (35%)	151 **	100% **	8
Water Use Efficiency	Environmental	22 (29%)	121	81%	5
HACCP Compliance Rate	Food safety	19 (25%)	28	19%	6
Global Diet Quality Score (GDQS)	Nutrition	15 (20%)	5	3%	4
AMR Prevalence in food chain isolates	One Health	12 (16%)	41	27%	3
Zoonotic disease incidence attributable to food	One Health	9 (12%)	67	45%	4

* Post-Harvest Loss Rate LMIC coverage (*n* = 102; 68% availability rate) is based on the FAO Food Loss Index (SDG indicator 12.3.1a) data and modelling methodology [40]. ** Foodborne Disease DALYs are available for all WHO member states through the WHO Global Health Observatory but rely on modelled estimates rather than primary surveillance data in most LMICs.

**Table 6:** Core Indicators for LMIC Monitoring.

Dimension	Indicator	Level	Data Source
Nutrition	GDQS	Individual	Nutrition surveys
Nutrition	MDD-W	Individual	DHS surveys
Food access	FIES	Household	FAO
Food safety	Foodborne disease DALYs	National	WHO
Food safety	HACCP compliance rate	Market	National food safety agencies
Sustainability	Cost of a healthy diet	National	FAO
Sustainability	Post-harvest loss rate	Supply chain	FAOSTAT
Sustainability	Water use efficiency	National	FAOSTAT
One Health	AMR prevalence in food chain isolates	National/supply chain	WHO GLASS; FAO/OIE AMR surveillance
One Health	Zoonotic disease incidence attributable to food	National	WHO GHO foodborne disease burden estimates
One Health	Pesticide residue exceedance rate in fresh produce	Market/supply chain	National food safety authority; Codex MRL compliance data

**Table 7:** Comparative Analysis of Existing Food System Monitoring Frameworks and the Proposed Integrated Framework for LMICs.

Feature	FAO Monitoring Systems	Food Systems Dashboard	Proposed Framework
Nutrition Indicators	Yes	Yes	Yes
Food Safety Indicators	Limited	Partial	Comprehensive
Sustainability Indicators	Partial	Yes	Yes
One Health Integration	No	Limited	Explicit
Indicator Selection Guidance	No	No	Yes
LMIC-specific Prioritization	Limited	Limited	Explicit
Policy Actionability Assessment	No	No	Yes
LMIC indicator selection decision tool	No	No	Yes
Informal sector coverage	No	Limited	Explicit

## Data Availability

No new data were created or analyzed in this study. Data sharing is not applicable to this article.
